# Reliability of foot posture index (FPI-6) for evaluating foot posture in patients with knee osteoarthritis

**DOI:** 10.3389/fbioe.2023.1103644

**Published:** 2023-01-18

**Authors:** Yi Wang, Zehua Chen, Zugui Wu, Junyi Li, Congcong Li, Jiaman Yang, Weijian Chen, Zixuan Ye, Xinxin Shen, Tao Jiang, Wengang Liu, Xuemeng Xu

**Affiliations:** ^1^ The Fifth Clinical College of Guangzhou University of Chinese Medicine, Guangzhou, China; ^2^ The Orthopedics Hospital of Traditional Chinese Medicine Zhuzhou city, Zhuzhou, China; ^3^ Guangdong Provincial Second Hospital of Traditional Chinese Medicine, Guangzhou, China

**Keywords:** foot posture, FPI-6, reliability, knee osteoarthritis, biomechanics

## Abstract

**Objective:** To determine the reliability of FPI-6 in the assessment of foot posture in patients with knee osteoarthritis (KOA).

**Methods:** Thirty volunteers with KOA (23 females, 7 males) were included in this study, assessed by two raters and at three different moments. Inter-rater and test-retest reliability were assessed with Cohen’s Weighted Kappa (Kw) and Intraclass Correlation Coefficient (ICC). Bland-Altman plots and respective 95% limits of agreement (LOA) were used to assess both inter-rater and test-retest agreement and identify systematic bias. Moreover, the internal consistency of FPI-6 was assessed by Spearman’s correlation coefficient.

**Results:** FPI-6 total score showed a substantial inter-rater (Kw = .66) and test-retest reliability (Kw = .72). The six items of FPI-6 demonstrated inter-rater and test-retest reliability varying from fair to substantial (Kw = .33 to .76 and Kw = .40 to .78, respectively). Bland-Altman plots and respective 95% LOA indicated that there appeared no systematic bias and the acceptable agreement of FPI-6 total score for inter-rater and test-retest was excellent. There was a statistically significant positive correlation between each item and the total score of FPI-6, which indicated that FPI-6 had good internal consistency.

**Conclusion:** In conclusion, the reliability of FPI-6 total score and the six items of FPI-6 were fair to substantial. The results can provide a reliable way for clinicians and researchers to implement the assessment of foot posture in patients with KOA.

## Introduction

Knee osteoarthritis (KOA) is a chronic disabling musculoskeletal condition affecting older adults, causing pain, reduction in quality of life, and physical disability (Davis, 2012). Additionally, it is the most common form of chronic joint disease and the foremost cause of lower limb disability among the elderly in the whole world (Zhang et al., 2010; Davis, 2012). Due to the aging of the population and the rising prevalence of obesity and multimorbidity, the prevalence of KOA is expected to rise (Reichenbach et al., 2020), imposing a substantial burden on the healthcare system (Cross et al., 2014). Etiology of KOA includes traumatic injury, genetic factors, age-related physiological changes, obesity, and poor joint biomechanics (Roos et al., 1995; Spector et al., 1996; Sharma et al., 2001; Toivanen et al., 2010). Moreover, poor biomechanics may be the cause of primary progressive KOA (Sharma et al., 2001).

Considering the important role of the foot in receiving and distributing forces during walking, the characteristics and mechanics of the foot, including static foot posture and dynamic foot function, may exert a significant impact on the musculoskeletal conditions of the lower extremities (Riskowski et al., 2011). From the biomechanical perspective, the degree of movement at the foot, subtalar and ankle joint affect the lower limb alignment as movement is transferred proximally (Gates et al., 2017). An excess of subtalar joint inversion/eversion could increase external/internal rotation of the tibia, which in turn is assumed to disrupt the normal mechanics of the tibiofemoral joint (Tiberio, 1987). These axial connections between the subtalar and the tibiofemoral joint demonstrate that the kinematics of the foot and ankle may have an impact on the both the transverse rotational and frontal measures about the knee (Gates et al., 2017). Thus, the foot is thought to play an important role in KOA (Lafortune et al., 1994). In fact, several studies have indicated that, in patients with KOA, any abnormalities (either as pronation or supination) in the foot posture could affect the force distribution throughout the entire lower extremity, including the knee joint, and is related to the misalignment of the knee joint (Paterson et al., 2015; Al-Bayati et al., 2018). Moreover, several studies have reported a more pronated foot posture in people with medial compartment KOA (Reilly et al., 2006; Reilly et al., 2009; Levinger et al., 2010). However, it remains unclear if abnormal foot posture contributes to the development of KOA, or whether progressive KOA leads to changes in foot posture as a compensatory mechanism (Levinger et al., 2012). A comprehensive assessment of foot posture could be important, therefore, for understanding the development of KOA and its conservative management (Reilly et al., 2009).

In fact, the measurement and classification of foot posture in clinical settings has become the central focus of lower extremity medicine and is now widely used to assess the risk of injury and monitor the efficacy of treatment (Mentiplay et al., 2013). There are many measures to quantify foot posture and function, including radiographic techniques, direct anatomical measures, footprint assessment, and dynamic laboratory analyses (Cavanagh et al., 1997; Williams and McClay, 2000). Laboratory gait analysis is still the gold standard, but the facilities to produce high-quality objective data are expensive, and the process of obtaining the data may be overly time-consuming for routine patient assessment (Redmond et al., 2006). Radiographic imaging is similarly demanding and has potentially harmful effects on human health due to the risk of exposing subjects to ionizing radiation (Cavanagh and Rodgers, 1987). As an objective clinical alternative, footprint-based measures are sometimes used, and although these have proved to be relatively reliable (Freychat et al., 1996), the relationship between these measures and dynamic functions is variable (Cavanagh and Rodgers, 1987; Hawes et al., 1992). In contrast, the Foot Posture Index-6 (FPI-6) is considered a quick, easy, inexpensive, and multi-segmental clinical quantification tool, which can assess the posture of the forefoot, midfoot and rearfoot from three planes, and can predict static and dynamic foot posture changes (Redmond et al., 2006; Nielsen et al., 2008). The FPI-6 consists of six evaluation criteria (Keenan et al., 2007). According to the quantitative results, the foot posture is divided into pronation position, neutral position, and supination position. Different from other instruments, FPI-6 contains most of the foot segments and three planes of motion (Redmond et al., 2006). Moreover, compared with other methods, there is a stronger correlation between FPI-6 and foot kinematics, indicating that FPI-6 provides a more complete description of foot posture (Redmond et al., 2006).

The FPI-6 has been extensively explored in a population of healthy people of different ages (Redmond et al., 2006; Keenan et al., 2007; Morrison and Ferrari, 2009). However, several studies assessing its inter-rater and test-retest reliability had conflicting results, with reliability differently reported as poor (Terada et al., 2014), moderate (Menz and Munteanu, 2005; Cornwall et al., 2008) and good (Morrison and Ferrari, 2009; Lee et al., 2015) reliability levels. To date, FPI-6 has not been widely used for foot assessment in patients with KOA, so data on the reliability of FPI-6 in assessing foot posture in KOA population is still lacking. Moreover, almost all previous studies only assessed the reliability of FPI-6 total score (Menz and Munteanu, 2005; Cain et al., 2007; Cornwall et al., 2008; Evans et al., 2012; Terada et al., 2014). Considering that the sum of different scores in individual items of FPI-6 may finally result in an equivalent total score, it remains unclear whether the raters of those previous studies were reliable in the FPI-6 individual items (Aquino et al., 2018). Notably, most KOA patients might present an abnormal foot posture owing to lots of factors including age, obesity, and alteration of the biomechanical axis, which would result in great variability of FPI scores, and subsequently affecting its reliability. Hence, it was necessary to investigate the reliability in KOA individuals. Given all of that, the aim of this study was to evaluate the inter-rater, test-retest reliability of FPI-6 total and individual scores for the assessment of foot posture in patients with KOA, and to provide the evidence basis for further application and promotion of FPI-6.

## Methods

### Study design

The study was performed between September–December 2021 in the orthopedic outpatient clinic of Guangdong Second Traditional Chinese Medicine Hospital. The study protocol was approved by the Ethics Committee of Guangdong Second Traditional Chinese Medicine Hospital (No. 2021(K58)) and registered in Chinese Clinical Trial Registry (Registration No. ChiCTR2100050269). Written informed consent was obtained from each participant.

## Participants

Gwet (Gwet, 2012) presented a method to estimate the sample size required in reliability studies, the expected observation agreement was .8, the chance agreement was .2, and the relative error was 40%. Then, the number of participants we need was 17 according to equation (36). In this study, a sample size of 30 participants was estimated. The inclusion criteria were: (I) age ≥50 years; (II) met the diagnostic criteria of the American College of Rheumatology (Altman et al., 1986); (III) Kellgren/Lawrence (KELLGREN and LAWRENCE, 1957) (K/L) grade ≥1; (IV) had symptoms in the unilateral or bilateral knee joints; (V) presence of predominantly medial compartment KOA; (VI) being able to remain in a static orthostatic position. The exclusion criteria were: (I) presence of other inflammatory rheumatic disease/arthritis; (II) had concomitant neurologic diseases, such as stroke, Parkinson’s disease, severe cardiovascular, respiratory, spinal cord injury, or other musculoskeletal diseases; (III) had a history of trauma resulting in structural deformity of the foot; (IV) not showing up for the retest.

### Procedure

Two investigators (Y W and ZH C) served as raters of the FPI-6 for both feet of 30 participants. Both raters had more than 3 years of clinical research experience in musculoskeletal aspects. Two raters attended a training course on the FPI-6 and communicated with each other during this training period for familiarization with FPI-6. In addition, the raters used FPI-6 in 30 feet before formal data collection in order to familiarize the assessment procedure. All participants were asked to stand, take a few steps forward and then to stand still, with arms by their side and looking forward. Given that the bias may be increased when successive measurements are made between the left and right feet, the first foot to be measured was always randomly chosen (Evans et al., 2012). Each item of FPI-6 was assessed and scored independently by each rater on a separate sheet. According to FPI-6 guidelines (Redmond, 2005), the raters were not allowed to see the contralateral feet of participants during the assessment of foot posture.

Six items of the FPI-6 were all assessed: (I) talar head palpation, (II) observation of curves above and below the lateral malleolus, (III) a bulge in the region of the talonavicular joint, (IV) eversion and inversion of the calcaneus, (V) congruence of the medial longitudinal arch, (VI) adduction and abduction of the forefoot in relation to the rearfoot. The score for each item was rated between −2 and +2, and the total score was between −12 and +12. The total score of ≥10 represented a highly pronated foot, 6 to 9 a pronated foot, 0 to 5 a normal foot, −1 to −4 a supinated foot and ≤−5 a high supinated foot (Redmond, 2005).

Two raters (Y W and ZH C) stood in the same position and successively independently evaluated both feet of participants. The participants remained in the same position while the second rater evaluated foot posture. The data measured by different raters on the same day were used to calculate the inter-rater reliability. To evaluate test-retest reliability, rater 1 (Y W) repeated data collection on the same participant 7 days after the first day of data collection. The raters were blinded to each other and their own data. Moreover, before further analyzing the data, we analyzed the left and right extremity data following the same methods used by Evans et al. (2003) to determine whether the left and right extremity data were suitable to be pooled. The results indicated that the left and right extremity data were suitable to pool and analysis. Therefore, both feet of participants were considered for analysis.

### Statistical analysis

All statistical analyses were performed with SPSS 26.0 for Windows (IBM, NY, US). Quantitative variables were presented as mean and standard deviation (SD), non-quantitative variables were presented as median and Interquartile range (IQR), and qualitative variables as counts and percentages. The normality was assessed firstly on collected data using the Shapiro-Wilks test. However, almost all data were not normally distributed, so we used non-parametric statistics.

We used Cohen’s Weighted Kappa (Kw) with linear calculation to assess FPI-6 inter-rater and test-retest reliability (Viera and Garrett, 2005). We performed Kw for each FPI-6 item, the total FPI-6 score, and the foot type classification data. The Kw values were interpreted as follows: 0 to .20 ‘slight’, .21 to .4 ‘fair’, .41 to .60 ‘moderate’, .61 to .8 ‘substantial’, and .81 or greater ‘almost perfect’ (Viera and Garrett, 2005). To allow comparisons with previous studies, we also calculated Intraclass Correlation Coefficient (ICC) for FPI-6 total raw score (i.e., not categorized). Inter-rater reliability was assessed using two-way random model, mean measures. And test-retest reliability was assessed using two-way mixed model, single measures (Koo and Li, 2016). The reliability was considered poor when the ICC <.4, fair when ICC ≥.4–≤ .59, good when ICC ≥.6–≤ .74 and excellent when ICC ≥.75 (van Geel et al., 2020). Moreover, the Bland–Altman plots obtained from the Medcalc software version 20.022 (Medcalc, Ostend, Belgium) and respective 95% limits of agreement (LOA) were used to assess the agreement and identify systematic bias for inter-rater and test-retest (Chen et al., 2019).

Additionally, the correlation between each item and the total scores was assessed to evaluate the internal consistency of FPI-6 using Spearman’s correlation coefficient. The consistency was acceptable when the correlation coefficient >.30 (Koo et al., 2020). Statistical significance was defined as *p* < .05.

## Results

### Participant characteristics

Thirty-three patients with KOA were recruited in the study, with a sample loss of three individuals who did not attend the retest. Therefore, the final sample consisted of 30 volunteers. Participant characteristics were shown in Table 1. The mean (SD) age of all participants was 67.70 (9.50) years and mean (SD) BMI was 23.56 (3.30) kg/m^2^. Most of the participants were women (76.7%). Fifteen participants (50%) had unilateral while the others (50%) had bilateral KOA. Most of the participants had a K–L grade of 2–3 (76.7%).

**TABLE 1:** Participant characteristics (*n* = 30).

Variable	Mean (SD) or n (%)
Age (years)	67.70 (9.50)
Height (cm)	160.37 (7.27)
Weight (kg)	60.98 (12.10)
BMI (kg/m^2^)	23.56 (3.30)
Gender (Female)	23 (76.7%)
Kellgren-Lawrence classification	
Grade 1	3 (10%)
Grade 2	7 (23.3%)
Grade 3	16 (53.3%)
Grade 4	4 (13.3%)
Limb involvement	
Unilateral	15 (50%)
Bilateral	15 (50%)

BMI, Body mass index; SD, Standard deviation.

### Inter-rater and test-retest reliability

The results of inter-rater and test-retest reliability were presented in Table 2. FPI-6 total score showed a substantial inter-rater and test-retest reliability. Additionally, FPI-6 total-score demonstrated inter-rater ICC of .94 (95%CI, .91-.97) and test-retest ICC of .96 (95%CI, .93-.97). The six items of FPI-6 demonstrated inter-rater and test-retest reliability varying from fair to substantial.

**TABLE 2:** Inter-rater and test-retest reliability of the FPI-6.

Variable	Median (IQR)			Inter-rater reliability			Test-retest reliability		
	Rater1	Rater2	Rater1^*^	Kw	95% CI	*p*-Value	Kw	95% CI	*p*-Value
Total FPI-6	2.50 (−1.00,5.00)	2.00 (−1.00,5.00)	1.50 (−1.00,4.75)	.66	.59-.72	.000	.72	.66-.79	.000
Item 1	1.00 (.00,1.00)	1.00 (.00,1.00)	1.00 (.00,1.00)	.55	.37-.72	.000	.58	.40-.75	.000
Item 2	1.00 (.00,1.00)	1.00 (.00,1.00)	.00 (.00,1.00)	.64	.48-.80	.000	.66	.52-.81	.000
Item 3	−1.00 (−1.00,0.75)	−.50 (−1.00,0.00)	−1.00 (−1.00,0.00)	.75	.64-.85	.000	.74	.64-.85	.000
Item 4	.00 (−1.00,0.75)	.00 (−1.00,0.00)	.00 (−1.00,0.00)	.33	.17-.48	.000	.40	.24-.55	.000
Item 5	.00 (.00,1.00)	1.00 (.00,2.00)	.00 (.00,1.00)	.74	.61-.87	.000	.78	.67-.90	.000
Item 6	1.00 (.00,2.00)	1.00 (.00,2.00)	1.00 (.00,2.00)	.76	.65-.87	.000	.69	.55-.83	.000

CI, Confidence interval; IQR, Interquartile range; Kw, Weighted Kappa; Item 1, Talar head palpation; Item 2, Curves above and below the lateral malleolus; Item 3, A bulge in the region of the talonavicular join; Item 4, Eversion and inversion of the calcaneus; Item 5, Congruence of the medial longitudinal arch; Item 6, Adduction and abduction of the forefoot in relation to the rearfoot; Total FPI-6, Foot Posture Index total score.

^*^ Rater 1 assessed again 7 days later.

### Agreement

**FIGURE 1:**
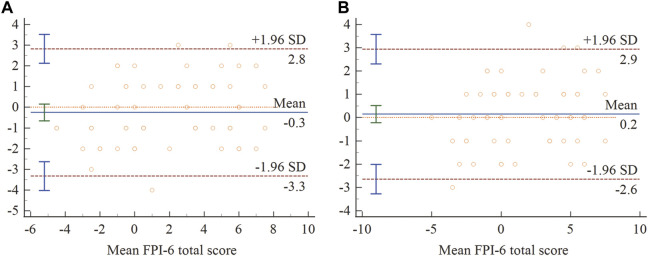
Bland–Altman plots of FPI-6 total score for inter-rater **(A)** and test-retest **(B)**. 95% limits of agreement marked with dotted (---) and mean difference marked with solid (−).

The Bland–Altman plots with the mean difference and 95% LOA for the level of inter-rater and test-retest agreement were shown in Figure 1. The mean difference of FPI-6 total score for inter-rater and test-retest was −.25 and .15, respectively, with the lower and upper limits of −3.33 to 2.83 and −2.64 to 2.94, respectively. These results indicated that there was little systematic bias and the acceptable agreement of FPI-6 total score for inter-rater and test-retest was excellent. Moreover, the correlation between each item and FPI-6 total score was shown in Table 3. The Spearman’s correlation coefficients of six items were all >.3 (*p* < .01). The results indicated that there was a statistically significant positive correlation between each item and FPI-6 total score.

**TABLE 3:** The correlations between each item and FPI-6 total score.

Items	Spearman’s correlation coefficient	*p*-Value
1. Talar head palpation	.51	<.01
2. Curves above and below the lateral malleolus	.59	<.01
3. A bulge in the region of the talonavicular join	.78	<.01
4. Eversion and inversion of the calcaneus	.63	<.01
5. Congruence of the medial longitudinal arch	.75	<.01
6. Adduction and abduction of the forefoot in relation to the rearfoot	.71	<.01

### Classification of foot type

The classification of foot type in the three moments of assessment was shown in Table 4. For the first assessment, rater 1 (Y W) classified 11 feet (18.3%) as being pronated, 31 (51.7%) as normal, 16 (26.7%) as supinated, 2 (3.3%) as highly supinated, and none as highly pronated. For the second assessment, rater 2 (ZH C) classified 13 feet (21.7%) as being pronated, 31 (51.7%) as normal, 16 (26.7%) as supinated, and none as highly pronated or highly supinated. Moreover, foot posture classifications made by rater 1 and 3 (test-retest) changed in 17 cases (*n* = 3 normal to pronated, *n* = 5 normal to supinated, *n* = 5 pronated to normal, *n* = 3 supinated to normal, and *n* = 1 highly supinated to supinated). Foot type classification data showed a moderate inter-rater reliability with Kw of .47 (95%CI, .31-.64) and a substantial test-retest reliability with Kw of .63 (95%CI, .47-.79), respectively.

**TABLE 4:** Classification of foot type in the three moments of assessment.

	Rater 2					
Rater 1	Highly pronated	Pronated	Normal	Supinated	Highly supinated	Total
Highly pronated	0	0	0	0	0	0
Pronated	0	7	4	0	0	11
Normal	0	6	20	5	0	31
Supinated	0	0	7	9	0	16
Highly supinated	0	0	0	2	0	2
Total	0	13	31	16	0	60
Rater 1^a^						
Rater 1	Highly pronated	Pronated	Normal	Supinated	Highly supinated	Total
Highly pronated	0	0	0	0	0	0
Pronated	0	6	5	0	0	11
Normal	0	3	23	5	0	31
Supinated	0	0	3	13	0	16
Highly supinated	0	0	0	1	1	2
Total	0	9	31	19	1	60

^a^ Rater 1 assessed again 7 days later.

## Discussion

To our knowledge, this study was the first to evaluate the reliability of FPI-6 in the assessment of foot posture in patients with KOA. The results indicated that the inter-rater and test-retest reliability of the total score of FPI-6 were substantial, and the inter-rater and test-retest reliability of the six items of FPI-6 were fair to substantial. Moreover, this study revealed that FPI-6 demonstrated good internal consistency. Therefore, FPI-6 could be considered as a reliable clinical evaluation tool to assess the foot posture of patients with KOA.

Foot posture affects the biomechanics of the entire lower extremity (Tong and Kong, 2013). Moreover, abnormal foot posture is closely related to many lower-limb injuries and the occurrence of various diseases (Tong and Kong, 2013). For instance, individuals with pronated feet are at high risk of falls or loss of balance during unilateral stance in functional activities; individuals with supinated feet may present disturbed postural control (Tsai et al., 2006). Additionally, there is evidence that abnormal foot posture interacts with the development of KOA (Levinger et al., 2012; Al-Bayati et al., 2018). Foot orthoses and insoles that can improve foot posture are also recommended for the prevention and treatment of KOA (Bannuru et al., 2019), so comprehensive foot posture evaluation contributes to understanding the development of KOA and choosing the best therapeutic intervention. Many indirect clinical techniques have been developed to evaluate foot posture, among which FPI-6 is favored by increasing clinicians since it can easily and quickly quantify the variation of foot position in various clinical settings (Scharfbillig et al., 2004). However, the reliability of FPI-6 varies (Menz and Munteanu, 2005; Cornwall et al., 2008; Terada et al., 2014; Lee et al., 2015), and there is a serious lack of data on the reliability of FPI-6 to assess foot posture in patients with KOA. Therefore, this study is of great significance for foot posture assessment and clinical intervention selection in patients with KOA.

We have found fifteen other studies on the reliability of FPI-6 (Menz and Munteanu, 2005; Cain et al., 2007; Morrison and Ferrari, 2009; Barton et al., 2010; Evans et al., 2012; Mentiplay et al., 2013; Lee et al., 2015; Langley et al., 2016; McLaughlin et al., 2016; Fraser et al., 2017; Aquino et al., 2018; Motantasut et al., 2019; Zuil-Escobar et al., 2019; Kirmizi et al., 2020; Martinez et al., 2021). Thirteen studies were conducted in healthy people, one in adults with patellofemoral pain syndrome and one in stroke patients. These studies indicated that the inter-rater reliability is fair to almost perfect, and the test-retest reliability varies from not reliable to excellent. The results of these studies were summarized in Table 5. However, the absence of data on the reliability of FPI-6 in KOA patients precluded comparison with other data of this population.

**TABLE 5:** Results from earlier reliability studies (FPI-6 total score).

Study	FPI type	Subjects	N	Intra-rater reliability	Inter-rater reliability	Test-retest reliability
Mentiplay et al. (2013)	FPI-6	Young, healthy males	30	ICC = .87		
Morrison and Ferrari, (2009)	FPI-6	Children	30		Kw = .86	
Cornwall et al. (2008)	FPI-6	Adults	46	ICC = .928-.937	ICC = .525-.655	
Lee et al. (2015)	FPI-6	Stroke patients	22	ICC = .870-.909	ICC = .807-.888	
Evans et al. (2012)	FPI-6	Healthy asymptomatic children	30	ICC = .93-.94	ICC = .79	
Cain et al. (2007)	FPI-6	Adolescent male Futsal players	76	ICC = .88	ICC = .69	
Aquino et al. (2018)	FPI-6	Adults	21		Kw = .57-.63; ICC = .79	Kw = .48-.65; ICC = .66-.69
		Older adults	19		Kw = .33-.41; ICC = .69	Kw = .04-.28; ICC = .41
Barton et al. (2010)	FPI-6	Adults with patellofemoral pain syndrome	15	ICC = .88-.97	ICC = .84-.92	
		Adults without patellofemoral pain syndrome	15	ICC = .92-.98	ICC = .79-.88	
Kirmizi et al. (2020)	FPI-6	Healthy adults	60/30	ICC = .945 (n = 60)	ICC = .575 (n = 30)	
Langley et al. (2016)	FPI-6	Adults	30	ICC = .93		
Zuil-Escobar et al. (2019)	FPI-6	Adults	20	K = .872	K = .829	
Fraser et al. (2017)	FPI-6	Healthy, young adults without history of ankle-foot injury	24		ICC = .81-.86	ICC = .81-.86
Martinez et al. (2021)	FPI-6	Adults	42	ICC = .9-.92	ICC = .91-.94	
Motantasut et al. (2019)	FPI-6	Asymptomatic adults	30	ICC = .98	ICC = .98	
McLaughlin et al. (2016)	FPI-6	Adults	83		ICC = .85-.86	

FPI-6, Foot Posture Index-6; ICC, Intraclass correlation coefficient; K, Kappa coefficient; Kw, Weighted Kappa.

Regarding inter-rater reliability, our study demonstrated that the Kw and ICC values of FPI-6 total score were .66 and .94, respectively. The Kw values of six items of FPI-6 vary from .33 to .76. Several studies on different age groups showed good inter-rater reliability for FPI-6 total score (lower than our study), which can be explained by the raters’ inconsistent familiarity with FPI-6 (Cain et al., 2007; Cornwall et al., 2008; Aquino et al., 2018). In addition, Kirmizi et al. (2020) only assessed the dominant foot, which may reduce the reliability while ensuring the independence of the data. In terms of test-retest reliability, the Kw and ICC values of FPI-6 total score were .72 and .96, respectively. The Kw values of six items of FPI-6 vary from .40 to .78. The test-retest reliability of FPI-6 total score is higher than those previously reported by either Aquino et al. (2018) and Fraser et al. (2017), the reason for the low retest reliability may be the low variability of foot posture for older adults (Allaj, 2018), since it increased the expected agreement due to chance (Viera and Garrett, 2005). The other reason might be explained by inconsistent measurement intervals.

The Bland-Altman plot was used to describe the mean score of the two assessments and the difference between them, and to assess whether there appeared a systematic bias for inter-rater and test-retest. Visual inspection of the Bland-Altman plots did not reveal any systematic bias between test and retest sessions. However, few studies on the reliability of FPI-6 had performed Bland-Altman analyses, which precluded comparison with published studies.

Moreover, the Spearman’s correlation coefficient was applied to determine the correlation between each item and the total score of FPI-6 to evaluate the internal consistency of FPI-6. The results showed that there was a statistically significant positive correlation between each item and the total score of FPI-6. In our study, the Spearman’s correlation coefficients of six items were all >.3 (*p* < .01). These results were similar to a published study where indicated the good internal consistency of FPI-6 in healthy participants or neuromuscular disease samples (Keenan et al., 2007).

In this study, sixty limbs of 30 participants were assessed at three different times. The results showed a moderate inter-rater reliability and a substantial test-retest reliability. In the analysis of a total of 180 foot postures, only 17 posture classifications changed between the first and the third evaluation. This result indicated the reproducibility of FPI-6 clinically, which was also demonstrated by Martinez et al. (2021) in their study. Changes in foot posture may be due to different positioning of participants during assessment process or to the scoring system of the tool, considering that a one-point difference can determine whether one is classified as being of one type verses another.

Additionally, the present study demonstrated that the total score of FPI-6 and the other five items had substantial reliability, except item 4. This can be explained by some of the limitations in the FPI-6 manual. For example, the FPI-6 manual has no example figure of intermediate scores and scores of −1 and 1, and the talonavicular joint region in the example figure of item 4 (Redmond, 2005) is displayed incorrectly, which was also mentioned by Aquino et al. (2018) and Kirmizi et al. (2020) in their discussion. Thus, the reliability of FPI-6 may be improved by adding figures for intermediate scores in the FPI-6 user guide and manual and correcting the example figure of item 4. In addition, previous studies (Cornwall et al., 2008; Langley et al., 2016) have shown that there existed a learning effect or at least an experience effect when using the tool. However, this effect was not observed in this study. There was a similar inter-rater and test-retest reliability for FPI total score (Kw = .65 and .72, respectively, for the first 30 feet examined, and .65 and .73, respectively, for the last 30 feet). This may be related to the fact that the raters of this study participated in the FPI-6 training course and practiced before the formal assessment.

However, the current study did present with some limitations. Firstly, the participants included were limited (*n* = 30), mostly female, and selected by means of a sample of convenience and did not cover the entire range that the FPI is designed to cover, that is, from the highly pronated to the highly supinated foot. Therefore, the complete range of scores for the correlation calculation was not obtained, reducing the strength of this calculation. Moreover, the results should be cautiously applied for men. Secondly, an additional limitation of this study was the inadequate refinement of FPI-6 categories. Due to the nature of FPI-6 (each of the six criteria has only five possible scores for all foot types), non-extreme foot types may have characteristics that are not easy to classify, making the selection of the appropriate criterion score less accurate. Therefore, further refinement of the definition of FPI-6 criterion scores may improve the reliability. Thirdly, the participants of this study involved unilateral or bilateral KOA patients. It is not clear whether this will affect the results of our study. Future studies should be conducted to further explore the effect of left and right feet on FPI-6 in unilateral or bilateral KOA patients. Additionally, the participants recruited in this study were not limited to patients with medial compartment KOA. Considering that medial compartment KOA was the most common type, it was necessary to further evaluate the reliability of FPI-6 in the assessment of foot posture in patients with medial compartment KOA.

In conclusion, the reliability of FPI-6 total score and the six items of FPI-6 were fair to substantial. The results can provide a reliable way for clinicians and researchers to implement the assessment of foot posture in patients with KOA.

## Data Availability

The original contributions presented in the study are included in the article/Supplementary Material, further inquiries can be directed to the corresponding authors.
